# [Expression of Concern] Anti-metastatic and differential effects on protein expression of epigallocatechin-3-gallate in HCCLM6 hepatocellular carcinoma cells

**DOI:** 10.3892/ijmm.2026.6014

**Published:** 2026-10-14

**Authors:** Yunjuan Zhang, Lawrence Owusu, Wei Duan, Tao Jiang, Shizhu Zang, Ayaz Ahmed, Yi Xin

Int J Mol Med 32: 959-964, 2013; DOI: 10.3892/ijmm.2013.1446

Following the publication of the above paper, it was drawn to the Editor's attention by a concerned reader that, for the cell invasion assay experiments shown in Fig. 2A on p. 962, panels 'b' and 'c' (showing treatment with 5 *μ*g/ml or 10 *μ*g/ml epigallocatechin-3-gallate respectively) contained an overlapping section of data, such that these data, which were intended to show the results from differently performed experiments, appeared to have been derived from the same original source.

The authors have been contacted by the Editorial Office to offer an explanation for the apparently incorrect assembly of data in Fig. 2, and we are awaiting their response. Owing to the fact that the Editorial Office has been made aware of potential issues surrounding the scientific integrity of this paper, we are issuing an Expression of Concern to notify readers of this potential problem while the Editorial Office conitnues to investigate this matter further.

